# Professor Draper’s Lecture

**Published:** 1851-11

**Authors:** 


### ECLECTIC DEPARTMENT,

### AND SPIRIT OF THE MEDICAL PERIODICAL PRESS.

---

*Professor Draper's Lecture.* — [We commence in this number of the Medical Gazette, the publication of a series of Physiological Lectures, with which our readers are to be favored by *Professor Draper*, of the N. Y. University. The first lecture is on the important subject of the " *Use of Fat in the Animal System.*" We venture to predict that our readers will find it highly instructive, while it presents views which are alike novel and interesting. As a writer and teacher, in his department, the Professor has no superior.] — *N. Y. Med. Gazette.*

Lecture I. — *On the Use of Fat in the Animal System.* By John W. Draper, M. D., Professor of Chemistry and Physiology in the University of New York.

There is deposited in certain parts of all animals a substance insoluble in water, fusible at a low temperature, combustible, and, though of variable constitution, known under the general designation of Fat.

I shall direct your attention to the nature and functions of this substance. It discharges an important duty in the economy.

Fats are secreted from the blood, in which they pre-exist, by the adipose cells, which sometimes occur sparingly scattered through the areolar tissues, or, when clustered, constitute the adipose. The primary form of these cells are spheroidal, though, as is often the case both in plants and animals, this form is departed from through the influence of pressure, and polygonal forms are assumed. Between the cells of adipose tissue a net-work of blood-vessels ramifies, for the double purpose of furnishing to the cells the fat they are to secrete, and likewise water; advantage being taken of the proverbial insolubility of all oily material in this liquid, and so long as the walls of the cells are kept moist, the contents cannot escape by transudation.

The adipose tissues occupy an intermediate position between the tissues that are constant and those that are variable. They do not necessarily exhibit that extreme proneness to change, so characteristic of the muscular or nervous. With some insignificant exceptions, which will be discussed hereafter, no oily substance ever escapes from the system until it has undergone change. These bodies being insoluble in water, cannot be removed in the urine.

It is not alone in animals, but also in plants, that we find fat. In the leaves of various grasses, in seeds, and fruits, it can be detected, by resorting to proper chemical processes. In those articles that are used as food by the herbivora, it constitutes a very appreciable part. One hundred pounds of indian corn contain about nine pounds of a thick oil, and one hundred pounds of dry hay contain about two pounds of fat.

What is the purpose for which nature resorts to this substance? I may answer that question by asking another. Why do men resort to it? Why do they go in ships and brave the winter of the Polar Seas, encountering the perils of the whale fishery? Why, in some parts of the country, are animals raised, as much for their fat as their flesh? What is the object of all those inventions which transmute the lard of the hog into a pure and cleanly body, approaching in quality spermaceti or the wax of the bee? It is for the purpose of availing ourselves of the combustion of this tribe of bodies, which experience has shown are the best of all sources of heat. Fat is burnt in lamps and candles, because it is the most compendious source from which a high temperature can be obtained. Nature resorts to the combustion of this substance in the interior of the system, for the same reason that we do in domestic economy.

The constitution of the common fats is that they contain carbon, hydrogen, and oxygen; the two former in great excess. During their oxydation a very large amount of heat is set free, because the heat-giving powers of hydrogen are brought into operation. When a fat burns, if there be an abundant access of air, the carbon turns into carbonic acid, and the hydrogen into water; but if the supply of air be limited, and this is a remark which should be borne in mind from its constant physiological application, the hydrogen burns away first and leaves the carbon. In our experiments, we often witness this; it gives origin to the dense black soot or smoke that arises from smoky lamps. When the combustion of an oil or fat in the system is complete, the products arising, carbonic acid and the vapor of water, are so constituted that they can escape through the lungs; and advantage is taken of this incident to effect the grand process of the introduction of atmospheric air. These bodies thus ministering to the functions of respiration, we speak of them as elements of respiratory food.

As was formerly shown, the fatty substances in the cases that more specially interest us, may be regarded as being composed of stearine and margarine, which are of a solid consistency, and oleine, which is liquid. We have seen that a rough analysis of an oily body may be made by submitting it to a certain degree of cold, when the solid parts congeal, and the fluid oleine may be separated. Or, the same may be accomplished by pressure, between folds of blotting paper, the paper becoming imbued with the liquid oil, and the solid matter remaining.

All these bodies, as might be inferred from their composition, have a very intense affinity for oxygen. Articles imbued with oil, as cotton, or flax, often take fire spontaneously. The oils in which this change most promptly takes place, assume a hard and resinous consistency, and are known as drying oils.

Two opinions have been entertained respecting the origin of the fat thus deposited in the tissues of animals. 1st. That it is manufactured in the system by certain vital or chemical metamorphoses from the food, in which it is not found to any great extent. 2d. That it is simply extracted from the food, in which it occurs naturally, being fabricated, in the first instance, by plants.

There are many facts which seem to show that fatty bodies can be formed from other organized substances. Several years ago it was discovered, on opening one of the burying grounds in Paris — the Cemetery of the Innocents — for the purpose of removing the dead bodies that had accumulated there, that all those which were below a certain depth had become converted into a fatty substance, now known under the name of adipocere. The muscles, the hair, the brain of these bodies appeared to have been entirely changed, giving origin to this substance, which has received its name from a resemblance it possesses to wax and fat.

A similar case occurred in this city. A female, who had been buried for nineteen years, was exhumed, and found to have passed into the condition in question. The whole body, so far as external appearances went, was completely preserved, being transformed into adipocere, and that without any sensible loss of substance. The features were perfect. In some places there were marks of the pressure of the funeral clothes — a piece of lace or other such ornament leaving its trace on the skin. The friends of the deceased had come to a very satisfactory conclusion as to the cause of the event. They told me that, the day before her death, the old lady had been engaged in pickling cucumbers, and was repeatedly seen immersed up to the elbows in the vinegar she was using. They therefore inferred that she had carried the thing too far, and pickled herself as well as the cucumbers!

A second instance of the apparent formation of fat is presented wherever fibrine is acted on by nitric acid, dissolution takes place, nitrogen is evolved, and a fatty substance is produced.

A third instance is in the preparation of oxalic acid from starch. The starch being digested in nitric acid, there is separated an oily body. These are some of the most prominent cases of the apparent artificial production of fat.

But, as respects the case of the Cemetery of the Innocents, and the production of adipocere generally, Chevreul has established its nature by showing that adipocere contains the same constituents as human fat, partially saponified by ammonia. A mass of flesh placed in a current of water, will, under certain circumstances, change into adipocere, but not more truly fatty matter can be obtained in this way, than could have been extracted directly from the flesh by the action of sulphuric ether. So we conclude that, whenever the change takes place, it is not a production or generation of fat, but the muscular and other tissues decaying away, the fat is simply set free, and becomes saponified by the ammonia arising during the putrefaction.

Next, as respects the cases of flesh-fat and starch fat, experiment shows that not more fat can be extracted in those cases than can be dissolved out by the aid of ether.

From these observations you will gather that, while it is not denied that fatty substances can arise from the metamorphosis or change of other organic bodies, as in the formation of butyric acid from sugar, the instances just brought forward by no means establish that position.

In a former lecture it was stated, that both waxes and fats occur in the leaves of plants. These substances possess such a relation to one another, that all the oily bodies may arise in succession from wax by a series of partial oxydations. Under the influence of the sunlight, the leaves effect the decomposition of carbonic acid, causing its oxygen to be evolved and its carbon to be fixed in their tissues. There can be little doubt that one of the starch family of bodies is the first to make its appearance. The formula of those bodies shows that, by partial oxydation, they can be converted into fat, a result that we witness every summer. The sap which has a sweet taste in the stem, loses its sweetness in proportion as oily matters form in the fruit.

From plants, animals derive the oleaginous substances they fix in their tissues. The vegetable world obtains them from the carbonic acid and water of the air,— the animal returns them back to the atmosphere as carbonic acid and water again. And, indeed, in this manner, all the carbonaceous atoms of which our bodies are composed, vibrate as it were backward and forward from the inorganic to the organic world. Now they reside in the air, and are tossed about by winds and currents — now they are organized as vegetable form, and, after serving awhile for the sustenance of animals, are cast back by processes of oxydation into the atmosphere, to run their race again.

The general mode of accumulating fat is by collection from the food, both in the case of carnivorous and also herbivorous animals,— the food in which it occurs most commonly to a sufficient extent. But the animal system can, when forced thereto, transmute both starch and sugar into the condition of oil, in the process of duodenal digestion.

I have so often incidentally referred to the physiological uses of this important body, its destruction by oxydation in the interior of the system, for the purpose of sustaining animal heat, so often pointed out the great superiority it possesses over other bodies in this respect, by reason of the large amount of hydrogen it contains, that it is scarcely necessary to dwell on those points in detail. In fevers, where there is an abstinence from food, we see how quickly the fat disappears, a general emaciation setting in, and from those deposits where it has been so carefully stored this combustible body is removed.

For the accumulation of fat, whether it be incidental, as in the human species, or purposed, as in the preparation of cattle for the market, there are obviously two conditions. The accumulation will depend — 1st. On the quantity of fat presented in the food. 2d. On the slowness of its consumption in the system. Now there are several circumstances which bear on this latter condition, and which here require to be pointed out.

1st. Whatever checks the respiratory process, or the introduction of oxygen into the system, will aid in the deposit of fat. Quick respiration implies quick oxydation; for the air introduced must have its affinities satisfied. To promote the accumulation of fat, an animal must be so situated that its respiration shall be slow.

2d. The higher the surrounding temperature the less is the loss of heat from the body by radiation and contact of the air, and the system is not required to develope so much heat, and consequently the destruction of fat is less. A high external temperature tends, therefore, to the accumulation of this body.

3d. Rest, or quiet. All movements taking place in the muscular tissues tend to the acceleration of the respiratory act. A man runs, and he quickly begins to pant. Large quantities of air are introduced, and the destruction of fat is the consequence. For this reason, of all the conditions under which an animal can be placed, sleep is by far the most favorable for the accumulation of fat. The respiration is tranquil and slow, there is a great freedom from muscular exertion, and usually the temperature is higher than when we are exposed in the pursuits of active life to the open air.

These things have been long understood by persons interested in the fattening of animals before their significance was detected by physiological chemistry. In certain places, where an inordinate obesity is given to animals for special purposes, each of these conditions is carefully observed. When geese are fattened for the sake of their livers, a delicacy much sought after by epicures, the process is to cram the bird with as much Indian corn, or other oily food, as possible, to tie its wings and feet, to ensure quiet, to place it in the chimney corner, or other warm situation, where the temperature is pretty high. Under these extraordinary conditions the bird sleeps profoundly, breathes slowly, introduces little air, destroys little fat. But the absorbents are busily engaged in taking it up, and an amazing accumulation is effected at last.

We sometimes see at agricultural exhibitions, hogs in a state of prodigious fatness. The form of the animal is altogether gone; if its feet touch the ground they are of no use as organs of locomotion; the snout barely projects beyond the rotundity of the face; the tip of the tail looks as if it were at the bottom of a pit. This forced condition of things has been produced by resorting to the precepts just laid down. The animal has been kept in a dark, warm place, crammed with oily food, in quiet, and asleep. Everything is done to lower the respiratory process, and abate the destruction of fat.

When we come to discuss the functions of the liver, we shall find that the secretion of that gland, the bile, stands in a certain relation to the respiratory function, — bile, the predominating constituents of which carbon, hydrogen, sulphur, are all combustible bodies. In cases where there is an interference with the respiratory functions, as in phthisis, and where less oxygen than usual is introduced into the system, these combustible bodies cannot be got rid of in the usual way, by converting them into carbonic acid, water, &c., and a reflected action is thrown upon the liver, which, unable to discharge its duty, often becomes engorged with fat. In this respect the condition is not unlike that artificially produced in the goose, as above mentioned.

I am persuaded, also, that these things are intimately connected with those embarrassments of the action of the liver, and hepatic diseases generally, which are so constantly encountered in hot climates, and in the warmer portions of our own country, in the hot seasons of the year. The high temperature of the surrounding air, often at a point near that of the animal system, prevents any great loss of heat either by radiation or by contact; the dew point, too, is commonly very high, and loss of heat by evaporation goes down to a minimum. In this semi-febrile condition, a man instinctively abstains from every thing that can raise his temperature, he avoids violent or perhaps even moderate exercise, he sleeps in the heat of the day, and, as far as he can, diminishes the activity of the respiratory functions, and the quantity of air introduced. But as the consequence of this, the lungs are unable to discharge their appointed duty, an embarrassment is thrown on the liver, the carbon and hydrogen, since they can be no longer burnt, fall under the action of that gland, which is overtaxed with the unnatural task.

It is for this reason that men in warm climates instinctively abhor all oily and fatty food, and choose fruits and watery diet. In these the amount of combustible materials is small — the use of them, therefore, leads to the evolution of little heat, and the disturbance I am dwelling on is to an extent avoided. How different with the man who lives in the cold north regions; an orange or pine apple is but a poor temptation to a Laplander or Esquimaux. He wants tallow and train oil. The cold air that surrounds him keeps his temperature down, so that he has hard work to keep it up. He wraps his greasy person in furs of the warmest kind, and consoles himself with the belief that in another and happier world the righteous shall feed on the blubber of whales.

What then, gentlemen, is the result at which we arrive from a full consideration of the subject? We conclude that man and all other animals under ordinary circumstances find in their food all the fat they require; that these have been made in plants by the all-pervading influence of the sun; but under special circumstances, we are constrained to admit, that if fat does not occur in the food to an extent sufficient for the wants of the system, the system by resorting to processes of subdivision, which we can artificially imitate, can manufacture it; that introduced by the lacteals, but not by the veins, the fats are either destroyed by gradual oxydation for the production of heat, one fat after another appearing in succession as these partial oxydations go on, and carbonic acid and water being developed at last, — or the excess is stored up in the adipose tissues for the future wants of the system, or, in the female, it passes in the secretion of the mammary gland, and is a constituent of milk. But whether it is thus stored up or thus secreted, its final duty is the same; it is to be burnt for the sake of the caloric it can evolve, and thus translated into carbonic acid and water, is restored to the atmospheric air, ready, under the influence of the sunshine, to be metamorphosed by plants back again into fat.

From these general views we now descend to particulars, and I shall proceed to offer you rigorous proof that both in herbivorous and carnivorous animals the fat deposited is not made in the stomach, but collected from the food. We shall then examine the system followed on a great scale by those who are interested in the fattening of cattle for the market, and also the production of milk, one of the main constituents of which is butter; this will furnish us with striking illustrations of the principles under consideration. Next, we shall see how all oily bodies, when once introduced into the system, begin to undergo change, and evolve caloric, and how, in order to regulate and control this, a special mechanism is resorted to, in which the cutaneous and respiratory surfaces and the malpighian bodies of the kidney discharge an important duty. — *N. Y. Med. Gazette.*

